# Phylogeography of Two Enigmatic Sulphur Butterflies, *Colias mongola* Alphéraky, 1897 and *Colias tamerlana* Staudinger, 1897 (Lepidoptera, Pieridae), with Relations to *Wolbachia* Infection

**DOI:** 10.3390/insects14120943

**Published:** 2023-12-13

**Authors:** Nazar A. Shapoval, Alexander V. Kir’yanov, Anatoly V. Krupitsky, Roman V. Yakovlev, Anna E. Romanovich, Jing Zhang, Qian Cong, Nick V. Grishin, Margarita G. Kovalenko, Galina N. Shapoval

**Affiliations:** 1 Department of Karyosystematics, Zoological Institute, Russian Academy of Sciences, Universitetskaya Nab. 1, 199034 St. Petersburg, Russia; 2 Photonics Department, Centro de Investigaciones en Optica, Lomas del Bosque 115, Leon 37150, Mexico; kiryanov@cio.mx; 3 Department of Entomology, Biological Faculty, Lomonosov Moscow State University, Leninskie Gory, GSP-1, korp. 12, 119991 Moscow, Russia; nephrurus@yandex.ru; 4 Severtsov Institute of Ecology and Evolution, Russian Academy of Sciences, Leninsky Pr. 33, 119071 Moscow, Russia; 5 Department of Ecology, Altai State University, Lenina Pr. 61, 656049 Barnaul, Russia; yakovlev_asu@mail.ru; 6 Institute of Biology, Tomsk State University, Lenina Pr. 36, 634050 Tomsk, Russia; 7 Resource Center for Development of Molecular and Cellular Technologies, St. Petersburg State University, Universitetskaya Nab., 7/9, 199034 St. Petersburg, Russia; aromanovich@gmail.com; 8 Department of Biophysics, University of Texas Southwestern Medical Center, Harry Hines Blvd. 5323, Dallas, TX 75390-9050, USA; jingzhang.first@gmail.com (J.Z.); qian.cong@utsouthwestern.edu (Q.C.); grishin@chop.swmed.edu (N.V.G.); 9 Department of Biochemistry, University of Texas Southwestern Medical Center, Harry Hines Blvd. 5323, Dallas, TX 75390-9050, USA; 10 Eugene McDermott Center For Human Growth & Development, University of Texas Southwestern Medical Center, Harry Hines Blvd. 5323, Dallas, TX 75390-9050, USA; 11 Research and Methodological Department of Entomology, All-Russian Plant Quarantine Center, Pogranichnaya 32, 140150 Bykovo, Russia; bush_zbs@mail.ru

**Keywords:** *Colias*, DNA barcoding, Lepidoptera, molecular taxonomy, PCR screening, *Wolbachia*

## Abstract

**Simple Summary:**

The genus *Colias* Fabricius, 1807 is a taxonomically challenging group of butterflies. Many *Colias* taxa display a high level of intraspecific variation in wing pattern and are weakly differentiated with respect to genitalia structure; therefore, a conclusion on their status as a distinct species, subspecies or local form can be controversial. In such cases, it is crucial to conduct a comprehensive analysis based on various phylogenetic and biogeographical approaches and a large-scale sampling dataset in order to resolve existing taxonomic and nomenclatural problems. In the present study, we focused on two enigmatic *Colias* taxa of unclear taxonomic status, *Colias mongola* Alphéraky, 1897 and *Colias tamerlana* Staudinger, 1897, restricted in distribution to western Mongolia, northwestern China and the south Siberian part of Russia. Here, we conducted a DNA barcode-based analysis that revealed complicate genetic pattern with several differentiated haplotypes clustered in four distinct haplogroups. In addition, we found a strong correlation between a specific mitochondrial haplogroup and *Wolbachia* infection, suggesting that *Wolbachia* endosymbionts may have played an essential role in the biology and diversification of the taxa in question and the genus *Colias* as a whole.

**Abstract:**

The genus *Colias* Fabricius, 1807 includes numerous taxa and forms with uncertain status and taxonomic position. Among such taxa are *Colias mongola* Alphéraky, 1897 and *Colias tamerlana* Staudinger, 1897, interpreted in the literature either as conspecific forms, as subspecies of different but morphologically somewhat similar *Colias* species or as distinct species-level taxa. Based on mitochondrial and nuclear DNA markers, we reconstructed a phylogeographic pattern of the taxa in question. We recover and include in our analysis DNA barcodes of the century-old type specimens, the lectotype of *C. tamerlana* deposited in the Natural History Museum (Museum für Naturkunde), Berlin, Germany (ZMHU) and the paralectotype of *C. tamerlana* and the lectotype of *C. mongola* deposited in the Zoological Institute, Russian Academy of Sciences, St. Petersburg, Russia (ZISP). Our analysis grouped all specimens within four (HP_I–HP_IV) deeply divergent but geographically poorly structured clades which did not support nonconspecifity of *C. mongola*–*C. tamerlana*. We also show that all studied females of the widely distributed haplogroup HP_II were infected with a single *Wolbachia* strain belonging to the supergroup B, while the males of this haplogroup, as well as all other investigated specimens of both sexes, were not infected. Our data highlight the relevance of large-scale sampling dataset analysis and the need for testing for *Wolbachia* infection to avoid erroneous phylogenetic reconstructions and species misidentification.

## 1. Introduction

*Colias* Fabricius, 1807 (Lepidoptera, Pieridae) is one of the largest pierid genera, with approximately 90 described species [1,2,3,4]. The genus has a predominantly Holarctic distribution, with a few species occurring in the Afrotropical, Oriental and Neotropical biogeographical regions [4,5,6,7]. Although *Colias* butterflies are among the most spectacular and well-known Lepidoptera, which historically attracted much attention from researchers and collectors, their taxonomy is still poorly elucidated. *Colias* butterflies may exhibit a significant level of interspecific variation in the wing pattern. At the same time, many allopatric taxa traditionally treated as separate species may have very similar phenotypes. In addition, some taxonomically important characters, such as genitalia structures, commonly used for species delimitation in insects, are largely uniform in this group and do not possess reliable diagnostic features [1]. Consequently, the systematics and real taxonomic status (species, subspecies or interspecific forms) of many *Colias* taxa are a matter of debate.

**Figure 1:**
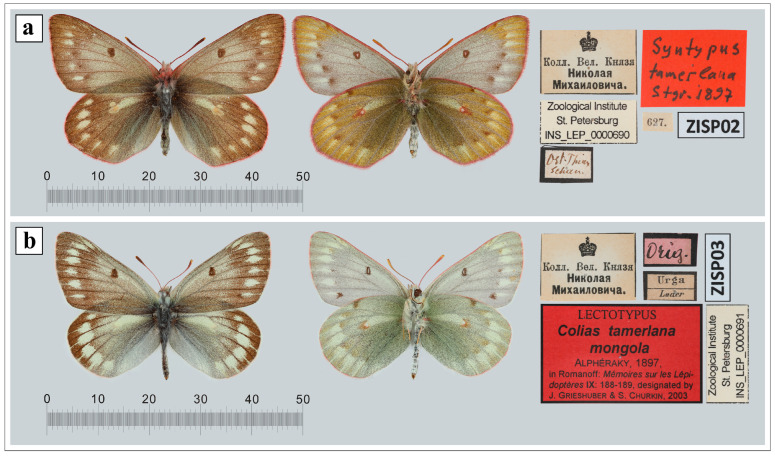
Type specimens of the *Colias* species deposited in the Zoological Institute, Russian Academy of Sciences, St. Petersburg, Russia (ZISP), barcoded in the present study: (**a**) paralectotype (female) of *C. tamerlana* Staudinger, 1897; (**b**) lectotype (male) of *C. mongola* Alphéraky, 1897.

**Figure 2:**
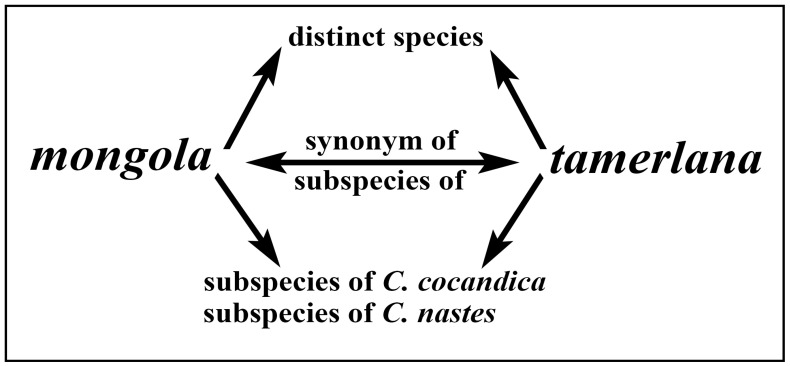
Relationships of *C. mongola*, *C. tamerlana* and the related taxa suggested by different authors (see text for explanation).

**Figure 3:**
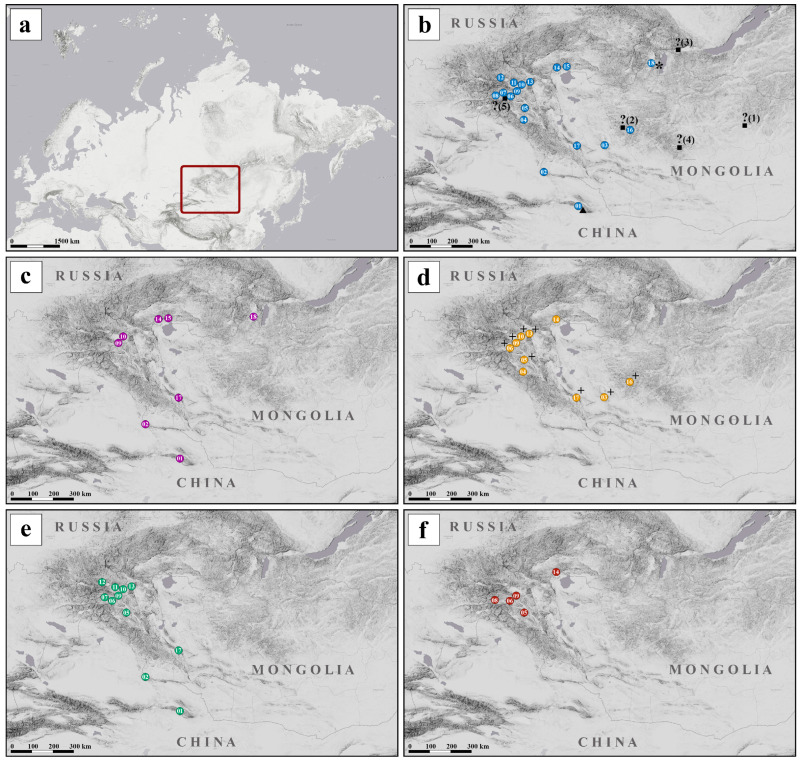
Maps showing location of the study area (red rectangle) (**a**), sampling localities of analyzed specimens of *C. mongola*/*C. tamerlana* (1–18) (**b**) and geographical distribution of *COI* haplogroups of *C. mongola*/*C. tamerlana* (**c**–**f***)*. Colours of circles correspond to revealed *COI* haplogroups; squares and question marks indicates suggested type localities of *C. mongola* (see text for explanation); triangle indicates type locality of *C. tamerlana*; asterisk indicates type locality of *C. tamerlana sidonia*; *Wolbachia* infected populations are indicated by “+”.

The issues mentioned above can be applied to *Colias tamerlana* Staudinger, 1897 (Figure 1a) and *Colias mongola* Alphéraky, 1897 (Figure 1b) distributed in western Mongolia, northwestern China and the south Siberian part of Russia [8,9]. Contradictory taxonomic status has been suggested for this pair of taxa in the literature, from synonyms or subspecies of several distinct species to separate species-level taxa (Figure 2). The former taxon was described as a separate species by Otto Staudinger in 1897 from China, Xinjiang, East Tian Shan, north of Hami, Karlik Shan Mts. [“im ostlichsten Thian-Schan-Gebirge (nordlich von Chamyl)”] [10]. The last one was described by Alphéraky as a variety of *Colias nastes* Boisduval, 1832 [*Colias nastes* B. var. *mongola* Alph. var. nova] in 1897 [11]. The type locality of *C. mongola* has been the subject of considerable debates and contradictorily interpreted in the taxonomic literature (Figure 3b). Korb [12] in his paper devoted to the taxonomy of *Colias cocandica* Erschoff, 1874 and allied taxa placed the type locality of *C. mongola* a few km south of Ulan-Bator (Mongolia). Grieshuber and co-authors [3] suggested that the type series of *C. mongola* originates from the vicinity of Uliastai (Mongolia), at a distance of ca. 750 km to the west of the locality indicated by Korb [12], but subsequently corrected the area to the SE Khangai Mts. [1]. Gorbunov [13] indicated the environs of the village Turan (Republic of Buryatia, Russia) as the type locality of *C. mongola*, an area located more than 500 km away from the localities suggested by Korb [12] and Grieshuber [1,3]. Finally, Weiss [14] erroneously mentioned the South Altai (Kuray, Chuya, Kobdo) as the type locality of *C. mongola*.

The nomenclatural priority of the name *C. tamerlana* over *C. mongola* [13] and the taxonomic status of these taxa are also disputable [2,12,13,14,15,16,17,18,19,20]. Staudinger treated *C. tamerlana* and *C. mongola* as synonyms [15]. Grieshuber and Lamas [2] pointed out weakly expressed morphological differences between the two taxa and speculated that molecular analysis could provide evidence for synonymizing *C. tamerlana* and *C. mongola.* Talbot [16] in his “*Lepidopterorum Catalogus*” placed *C. tamerlana* as synonym of *C. cocandica maja* Grum-Grshimailo, 1891 without providing further explanations. Korb [12], based on examinations of genitalia, concluded that *mongola* and *tamerlana* represent distinct species. Tshikolovets and co-authors [17] regarded *C. mongola* as a subspecies of *C. cocandica*. These two taxa are thought to be biogeographically isolated (the eastern limit of *C. cocandica* distribution range lies in SE Kazakhstan (south Dzungaria) and NW China (Borohoro Mts.)) [3]; thus, the conclusion of their conspecificity is rather doubtful. It should be noted that butterflies from the Lake Khovsgol area (Mongolia), currently considered as the easternmost known population of *C. tamerlana/mongola*, were originally referred to the much westerly distributed *C. cocandica* and were described as a subspecies of the latter, *C. cocandica sidonia* Weiss, 1968 [14]. Some authors regarded this taxon as a subspecies of *Colias nastes* Boisduval, 1832, without considering the relationships between *C. mongola* and *C. tamerlana* and their taxonomic status [18,19,20,21], while mentioning that *C. mongola* was formerly treated as a separate species.

To some extent, this assumption was based on the discovery of a large series of specimens, later described as *Colias nastes jacutica* Kurentsov 1970, which phenotypically somewhat resembles *sidonia,* the taxon that was provisionally considered a valid subspecies of *C. tamerlana/mongola.* In the original description [21], *Colias nastes jacutica* is interpreted as an intermediate link between *nastes* and *mongola* (sensu lato); yet, this assumption requires further confirmation (in particular, by applying molecular methods), given that *sidonia* and *nastes* are separated by more than 2500 km without any record for the species in the gap. The second subspecies of *C. tamerlana/mongola, C. mongola ukokana* Korb and Yakovlev, 2000, was described from the Ukok Plateau (Republic of Altai, Russia) [22], but currently *ukokana* is considered a synonym of the nominotypical *mongola* [1,2,3].

The correct delineation and identification of species is not always possible based on characters of external morphology alone, especially in such taxonomically challenging groups as *Colias* [23,24,25,26]. Over the last decades, the rapid development and implementation of new molecular techniques and approaches validated mitochondrial (e.g., DNA barcodes) and nuclear DNA markers as a useful and efficient tool for species identification, detecting and analyzing cryptic diversity, revealing interspecific and deep-level relationships and phylogenetic structure in taxonomically challenging groups of insects, solving long-standing taxonomic problems [23,27,28,29,30,31,32,33,34,35,36,37,38,39,40,41]. Despite the broad and constantly growing usage of DNA-based techniques in butterfly taxonomy and molecular systematics, surprisingly few studies focused, so far, on the genus *Colias* [6,42,43,44,45,46]. Moreover, all but a few research studies aimed at inferring *Colias* phylogeny or relating to molecular aspects of the genus were based on a limited set of taxa and/or poor specimen sampling. Some recent studies and our unpublished data suggested that morphologically well-differentiated *Colias* species may have nearly identical or even shared *COI* (cytochrome c oxidase subunit 1 gene) haplotypes. For instance, D’Ercole and co-authors [47] showed that all 22 North American *Colias* species shared at least one of the revealed barcode sequences with another species. Conversely, two or more deeply diverged mitochondrial lineages (e.g., DNA barcodes) may be found within the same species [42]. In such cases, imbalanced taxa sampling and/or testing only a small number of specimens per species could obscure the true phylogenetic relationships within the genus and lead to species misidentification and incorrect taxonomical conclusions. To overcome this problem, all *Colias* taxa should be analyzed in detail to ascertain their actual taxonomic status and avoid erroneous interpretations.

Here, we analyzed three nuclear genes, *Ca-ATPase* (sarco/endoplasmic reticulum calcium ATPase), *H3* (Histone h3) and *CAD* (Carbamoyl-Phosphate Synthetase 2, Aspartate Transcarbamylase and Dihydroorotase), as well as the barcoding region of the mitochondrial *COI* gene, for a large set of *C. mongola* and *C. tamerlana* samples, including, recovered for the first time, DNA barcodes of the century-old type specimens. We also conducted PCR screening for three *Wolbachia* genes of the collected *C. mongola* and *C. tamerlana* butterflies in order to reveal patterns of *Wolbachia* infection.

Based on a large-scale sampling dataset, our study aimed to (i) reveal the phylogeographic structure of *C. mongola* and *C. tamerlana*, (ii) clarify their taxonomic status, contradictory interpreted in the literature and (iii) shed light on the role of *Wolbachia* in evolutionary history and observed biogeographic patterns of the taxa in question.

## 2. Materials and Methods

### 2.1. Taxon Sampling

Ninety-one specimens of *C. mongola* and *C. tamerlana* were collected in various localities of western Mongolia, northwestern China and the south Siberian part of Russia (Figure 3a), covering the known distribution ranges of these taxa. All sampling sites were partitioned into eighteen populations (Figure 3b). Samples either were preserved in 96% alcohol or were kept dry for subsequent molecular analysis. The lectotype of *C. tamerlana* deposited in the Natural History Museum (Museum für Naturkunde), Berlin, Germany (ZMHU), the paralectotype of *C. tamerlana* and the lectotype of *C. mongola* deposited in the Zoological Institute, Russian Academy of Sciences, St. Petersburg, Russia (ZISP), four specimens of *C. tamerlana sidonia* from the type locality deposited in the Zoological Museum of the Moscow State University, Moscow, Russia (ZMMU) and three samples mined from BOLD database (http://www.boldsystems.org, accessed on 3 September 2022) were included in the sampling dataset. Thus, the final dataset included 101 specimens. The list of the specimens used for the molecular analysis with identification codes and collection data is given in the electronic Supplementary Materials (Table S1).

### 2.2. DNA Extraction

One leg from each specimen was taken for DNA extraction. For the samples more than 10 years old, the total genomic DNA was extracted using QIAamp DNA Investigator Kit (Qiagen, Venlo, The Netherlands), following the manufacturer’s protocol. For the specimens up to 10 years old, DNA extraction was performed using the CTAB-based method [48] with some modifications [39,49]. The segments were homogenized in CTAB buffer and digested with proteinase K (10 mg/mL) overnight at 56 °C. DNA was purified through successive ethanol precipitations and stored in dd H_2_O at −20 °C.

### 2.3. Molecular Markers, PCR Amplification and Sequencing

One mitochondrial (*COI*) and three nuclear (*Ca-ATPase*, *H3* and *CAD*) genes were used as molecular markers. A 658 bp fragment of the *COI* gene (mitochondrial DNA barcode) was amplified using LCO1490/HCO2198 [50] and LepF/LepR primer pairs [51]. In case standard lepidopteran barcode primers failed to yield a sufficient product, we amplified full-length barcode fragments using the primer pair combinations LepF/MH-MR1 + MH-MF1/LepR and LCO1490/MH-MR1 + MH-MF1/HCO2198 [52]. Primers CAD743nF/CADmidR, CADmidF/CAD1028R [53], H3aF/H3aR [54] and Ca-ATPase_F/Ca-ATPase_R [55] were used for nucDNA amplification and resulted in 847 bp fragment of the *CAD*, 328 bp fragment of the *H3* and 445 bp fragment of the *Ca-ATPase* genes, respectively.

The PCR amplifications were performed in a 15 µL reaction volume per sample. Each reaction contained 1 µL template DNA (ca. 10–50 ng genomic DNA), 0.9 µL of both forward and reverse primers diluted to a standard concentration of 10 µM, 3 µL of 5× ScreenMix (Evrogen, Moscow, Russia) and 9.2 µL of ddH_2_O. The temperature profile for *COI*, *CAD* and *Ca-ATPase* genes was as follows: initial denaturation at 95 °C for 5 min, followed by 35 cycles of denaturation at 94 °C for 30 s, annealing at 50 °C (*COI*)/55 °C (*CAD*, *Ca-ATPase*)/60 °C (*H3*) for 30 s and extension at 72 °C for 1 min 30 s, with a final extension at 72 °C for 10 min. The purified PCR products were subjected to further sequencing. Sequencing of the double-stranded product was carried out at the Research Resource Center for Molecular and Cell Technologies (St. Petersburg State University, St. Petersburg, Russia) using ABI 3500xL analyzer (Applied Biosystems, Waltham, MA, USA).

### 2.4. Processing and Sequencing of Old Type Specimens

To obtain *COI* barcodes of the century-old type specimens we followed the protocol described in detail by Li et al. [56]. In brief, a single leg was used for DNA extraction. The DNA isolation protocol was non-destructive: the entire leg was soaked in a DNA extraction solution overnight and was preserved after the extraction. Genomic libraries were constructed from total DNA and sequenced for 150 bp from both ends on Illumina HiSeq ×10 (Illumina, San Diego, CA, USA) in a pool with others. The sequences were demultiplexed to assign to each specimen by the index and indices removed. The barcodes were assembled using a reference sequence as a bait. All sequence reads matching the reference were mapped to it using DIAMOND [57]. The coverage of the barcode sequence is typically high (100 to 1000-fold or more), and overlapping reads resulted in an unambiguous sequence. Further details of experimental and computational protocols can be found in [56].

### 2.5. Detection of Wolbachia Endosymbionts

*Colias* specimens were screened for the presence of *Wolbachia* infection by amplifying three *Wolbachia* genes, *16S ribosomal RNA* (*16S*), *Wolbachia surface protein* (*wsp*) and *Filamentation temperature-sensitive protein Z* (*ftsZ*). We used *Wolbachia*-specific primer pairs, W-Specf/W-Specr [58], wsp81F/wsp691R [59] and ftsZ-F/ftsZ-R [60], amplifying ~ 396 bp fragment of the *16S RNA* gene, ~ 549 bp fragment of the *wsp* gene and ~ 510 bp fragment of the *ftsZ* gene (actual length of PCR fragments may vary, depending on the individual *Wolbachia* strain), respectively. The PCR amplifications were performed in a 15 µL reaction volume. Each reaction contained 1 µL template DNA (ca. 10–50 ng genomic DNA), 0.8 µL of both forward and reverse primers diluted to a standard concentration of 10 µM, 3 µL of 5× ScreenMix (Evrogen, Moscow, Russia) and 9.4 µL of ddH_2_O. The temperature profile for *16S*, *wsp* and *ftsZ* genes was as follows: initial denaturation at 95 °C for 5 min, followed by 40 cycles of 30 s denaturation at 95 °C, 1 min annealing at 50 °C and extension at 72 °C for 45 s, with a final extension at 72 °C for 5 min. Each PCR reaction contained two negative (PCR mix with ddH_2_O instead of DNA sample) and one positive (genomic DNA of a *Wolbachia*-infected *Colias* specimen, previously successfully amplified for *16S*, *wsp* and *ftsZ* genes) controls. PCR amplification was conducted twice for each specimen in order to avoid technical errors. To ascertain the presence/absence of *Wolbachia*, each PCR product was checked on 1% standard agarose gel. Our personal unpublished data suggests that the standard screening procedure for *Wolbachia* allows detecting infection in host specimens up to 30–35 years old but it sufficiency largely depends on the quality of the genomic DNA and storage conditions of the specimens. Thus, to avoid false-negative results, we excluded from the analysis samples collected more than twenty years ago (before the 2000s). Specimens positive for three *Wolbachia* genes were sequenced at the Research Resource Center for Molecular and Cell Technologies (St. Petersburg State University, St. Petersburg, Russia) using ABI3500xL Genetic Analyzer (Applied Biosystems, Waltham, MA, USA).

### 2.6. Molecular Data Analysis and Phylogenetic Reconstructions

All sequences were checked for errors, edited and aligned using Geneious v.8.1.6 [61] and BioEdit v.7.0.3 [62] software. Primer sequences were cropped. The final *COI* dataset (alignment length 658 bp) included 101 sequences of *C. mongola*/*C. tamerlana*. Sequences of pierids *Leptidea juvernica* (Linnaeus, 1758) and *Colias croceus* (Geoffroy, 1785) (GenBank accession numbers MT210323 and OR178497, respectively) obtained previously were included as an outgroup to root the phylogram. *COI* sequences were collapsed to unique haplotypes using online tool FaBox v.1.61 (https://birc.au.dk/~palle/php/fabox/, accessed on 5 November 2022) [63]. The concatenated nuclear dataset (alignment length 1620 bp) included sequences of 17 *C. mongola*/*C. tamerlana* specimens representing all revealed *COI* haplogroups. Phylogenetic reconstructions placed *C. croceus* within basal lineages of the genus *Colias*, thus it was selected as the outgroup to root the mitochondrial and nuclear phylograms [42,43]. Nucleotide substitution models for each dataset were estimated based on the Bayesian information criterion (BIC) using jModelTest v.2.1.10 [64]. The best-fitting models were as follows: JC for *COI* dataset and HKY+I for concatenated *Ca-ATPase*+*H3*+*CAD* nuclear genes fragments. Bayesian analyses were performed using the MrBayes v.3.2.7a software [65]. Parameters were estimated using two independent runs of 10 million generations each with four simultaneous chains (one cold and three heated). The sampling of trees and parameters was set to every 1000 generations. The first 10% of trees were discarded as burn-in prior to computing a consensus phylogeny and posterior probabilities. TRACER v.1.6 was used for checking the stationarity and convergence of Bayesian analyses between runs [66]. The consensuses of the obtained trees were visualized using FigTree v.1.4.4 (http://tree.bio.ed.ac.uk/software/figtree/, accessed on 5 November 2022). For the analysis of the phylogeographical structure of *C. mongola* and *C. tamerlana*, a median-joining haplotype network [67] was built using popART v.1.7 software [68]. Genetic distances among *COI* barcodes were calculated using MEGA v.7.0.14 [69]. The number of polymorphic and parsimony informative sites, the number of haplotypes, haplotype (*h*) and nucleotide (π) diversities were calculated in DnaSP v.6.12.03 [70]. DnaSP was further used to infer the demographic history of *C. mongola* and *C. tamerlana* with Tajima’s D [71], Fu and Li’s D [72], Fu and Li’s F [72] and Fu’s Fs [73] statistical tests for neutrality.

### 2.7. Molecular Characterization of Wolbachia

The BLAST algorithm implemented in NCBI (https://blast.ncbi.nlm.nih.gov, accessed on 20 July 2023) was used to search for sequence similarities in GenBank database with known DNA (BLASTN) sequences. We mined 43 *16S*, 74 *wsp* and 71 *ftsZ* sequences with the highest percentage identity match, which were included in the alignment datasets. In the final alignments, identical *Wolbachia* sequences of the same host species were limited to one record. To estimate relationships among *Wolbachia* alleles, phylogenetic analyses were conducted for each gene independently using the Bayesian inference (BI) approach, applying GTR+G (for *16S* gene fragment) and GTR + I +G (for *wsp* and *ftsZ* genes fragments) substitution models, as suggested by jModelTest v.2.1.7 [64]. All other parameters of the Bayesian analyses were the same as for *COI* alignment.

### 2.8. Data Availability

All sequences obtained for *COI*, *CAD*, *Ca-ATPase* and *H3 Colias* genes and for *16S*, *wsp* and *ftsZ Wolbachia* genes were deposited to GenBank under accession numbers OP946559–OP946652, OR178498–OR178501, OR178497 (*COI*), OQ192178–OQ192194 (*CAD*), OQ1921161–OQ192177 (*Ca-ATPase*), OQ192144–OQ192151 (*H3*), OQ155222–OQ155235 (*16S*), OQ192116–OQ192129 (*wsp*) and OQ192130–OQ192143 (*ftsZ*) and listed in the electronic Supplementary Materials (Table S2). Voucher specimens were deposited in the Department of Karyosystematics of the Zoological Institute of the Russian Academy of Sciences and private collections of A. Kir’yanov, A. Krupitsky, A. Marusov, A. Kurmaev, S. Churkin (Moscow, Russia), B. Khramov (St. Petersburg, Russia) and R. Yakovlev (Barnaul, Russia).

## 3. Results

### 3.1. Haplotypic Diversity of C. mongola/C. tamerlana

**Figure 4:**
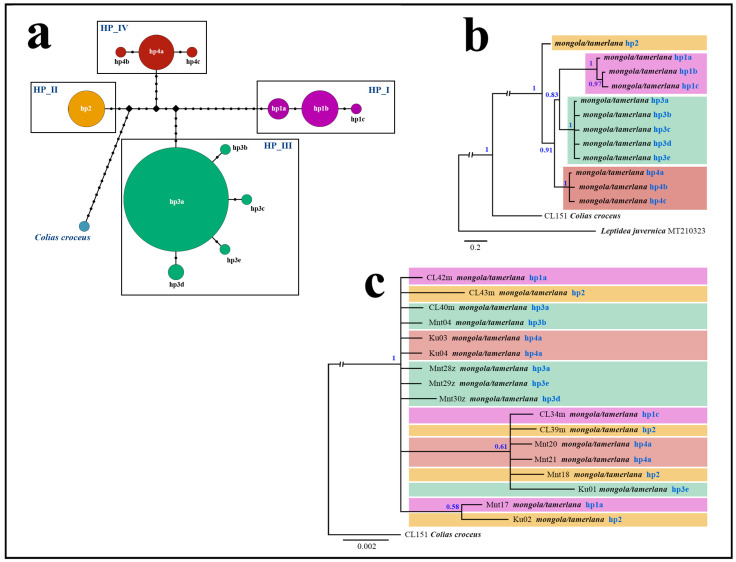
Phylogenetic patterns of *C. mongola*/*C. tamerlana* based on the analysis of mitochondrial (*COI*) and nuclear (*Ca-ATPase*, *H3* and *CAD*) markers. (**a**) Median-joining haplotype network illustrating relationships of the revealed *COI* haplotypes; mutations are shown as 1-step edge. (**b**,**c**) The Bayesian consensus trees of *C. mongola*/*C. tamerlana* inferred from *COI* sequences (**b**) and concatenated alignment of three nuclear markers (**c**); numbers at nodes indicate Bayesian posterior probabilities (PPs).

Haplotype analysis of a dataset of 101 *C. mongola* and *C. tamerlana* specimens revealed 12 *COI* haplotypes clustering in four distinct haplogroups (HP_I–HP_IV) (Figure 4a). Each haplogroup consists of one main haplotype and one to four satellites, differing from the main haplotypes in one nucleotide substitution. The only one exception is haplogroup HP_II, which consists of a single haplotype hp2. A low rate of genetic diversity ranging from 0% to 0.07% ± 0.05% was detected within each haplogroup. On the contrary, sequence divergence between haplogroups is relatively high (1.23% ± 0.41%–2.56% ± 0.62%), with a maximum p-distance of 2.89% ± 0.62% between the most divergent haplotypes (Table 1).

In general, haplotype distribution demonstrates no clear geographical structure: haplotypes of all four haplogroups commonly occur in sympatry; at the same time, they can be found in the geographically remote populations (Figure 3c–f). Twenty-six specimens clustered in three haplotypes of the haplogroup HP_I was shared by 9 populations. Seventeen specimens constituted a single haplotype of the haplogroup HP_II, which occurred in 10 sampling sites. Forty-two specimens were grouped into five haplotypes of the haplogroup HP_III and sixteen specimens constituted three haplotypes of the haplogroup HP_IV, which were found in 11 and 4 sampling sites, respectively. Four haplotypes, namely hp1b, hp2, hp3a and hp4a, were the most commonly observed haplotypes out of all screened individuals. On the contrary, haplotypes hp1c, hp3b, hp3c, hp4b and hp4c, consisting of only one specimen each, were found in single sampling sites. Detailed data on the distribution of the haplotypes among specimens and localities is given in Table 2.

### 3.2. Phylogenetic Analyses of Mitochondrial and Nuclear Markers

The Bayesian phylogenetic tree for *C. mongola*/*C. tamerlana COI* haplotypes displayed four strongly supported (PP = 1) lineages (Figure 4b), corresponding to four haplogroups revealed by haplotype network analysis. Within the *C. mongola*/*C. tamerlana* clade, a basal position is occupied by haplotype hp2, which is common and widespread across the geographical area studied. Further splitting recovers the clade containing specimens of the HP_IV haplogroup. This clade, in turn, appeared as a sister to the remaining two clusters, consisting of haplotypes hp3a–hp3e and hp1a–hp1c, respectively.

Combined analysis of three nuclear markers (*Ca-ATPase*, *H3* and *CAD*) resulted in an unresolved tree for *C. mongola*/*C. tamerlana* that failed to recover clades revealed by BI analysis of *COI* barcodes (Figure 4c). In general, nuclear markers demonstrate a very shallow divergence forming on the phylogenetic reconstructions two unsupported clades (PP = 0.58; PP = 0.61), each consisting of specimens bearing *COI* haplotypes of different haplogroups. Both intra-individual heterogeneities and single nucleotide substitutions were found in sequenced fragments of *Ca-ATPase*, *CAD* and *H3* nuclear genes among the 17 analyzed specimens of *C. mongola*/*C. tamerlana*. However, polymorphic sites were distributed inhomogeneously across the three genes fragments. The 847 bp fragment of the *CAD* was the most variable with 23 heterogeneities/substitutions found, whereas 445 bp fragment of the *Ca-ATPase* gene and 328 bp fragment of the *H3* gene were conserved, having four and one polymorphic sites, respectively. Detailed information on the nucleotide variability of the studied nuclear gene fragments is given in the electronic supplementary (Table S3).

### 3.3. Wolbachia Analysis

**Figure 5:**
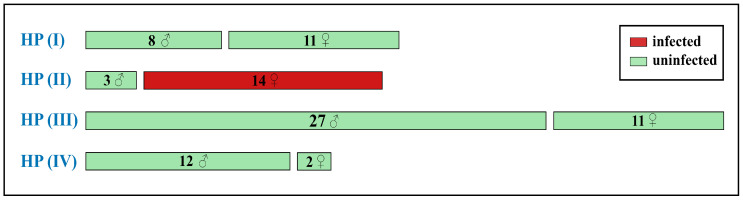
Infection rates of *Wolbachia* in 88 specimens of *C. mongola*/*C. tamerlana* analyzed in the present study. Values and symbols in each bar indicate the number and sex of individuals bearing *COI* haplotypes of certain haplogroup. Green and red bars indicate uninfected and infected specimens, respectively.

A total of 88 specimens of *C. mongola*/*C. tamerlana*, representing all four *COI* haplogroups recovered, was screened for the presence of the *Wolbachia* infection. Screening for three *Wolbachia* genes (*16S, wsp* and *ftsZ*) did not reveal any cases of dissimilar results (i.e., when a specimen was positive for one *Wolbachia* gene, but negative for another gene/genes). In total, 14 specimens out of 88 tested were scored positive for *Wolbachia* infection (prevalence: 16%). Our analysis suggested a sex-dependent congruence between a specific *COI* haplogroup and *Wolbachia* infection: all 14 infected specimens were females of the *COI* haplogroup HP_II comprising a single haplotype hp2. Three analyzed males of this haplogroup, as well as all other investigated specimens, were not infected (Figure 5). Our survey did not recover a specific geographical pattern of *Wolbachia* incidence: infected specimens were randomly found in the geographically remote populations (Figure 3d).

#### 3.3.1. Wolbachia Allele Identified in *C. mongola*/*C. tamerlana*

*Wolbachia 16S*, *wsp* and *ftsZ* genes were sequenced for all infected specimens (14 females) of *C. mongola*/*C. tamerlana*. These specimens were infected by a *Wolbachia* strain belonging to the supergroup B (grouping according to [74], which was designated as *w*Tam (name following the widely accepted abbreviation style [59,75]).

Comparison with *Wolbachia* alleles found in other *Colias* taxa (personal unpublished data) revealed that the *w*Tam strain isolated from *C. mongola*/*C. tamerlana* was most similar to one of the strains found in *C. palaeno* (Linnaeus, 1761), having, with the latter, identical *16S* and *ftsZ Wolbachia* sequences and differing in one nucleotide substitution in the *wsp* gene fragment. Infected *C. mongola*/*C. tamerlana* specimens share the *16S Wolbachia* sequence with several insect taxa, belonging to orders Lepidoptera (Pieridae, Nymphalidae, Tortricidae), Orthoptera (Acrididae), Hemiptera (Issidae, Lygaeidae) and *wsp* sequence with *Phengaris nausithous* (Bergsträsser, 1779) (Lepidoptera, Lycaenidae). The closely related *Wolbachia* alleles were found in Hemiptera families Aleyrodidae, Triozidae, Delphacidae, Aphididae and Liviidae for the gene *16S*; in insect orders Lepidoptera, Orthoptera, Hemiptera, Diptera, Hymenoptera and spider mites (Trombidiformes) for the gene *wsp*; and in Lepidoptera, Orthoptera, Hemiptera, Diptera, Hymenoptera, Coleoptera and Trombidiformes for the gene *ftsZ* (https://blast.ncbi.nlm.nih.gov, accessed on 20 July 2023).

#### 3.3.2. Phylogenetic Inferences

**Figure 6:**
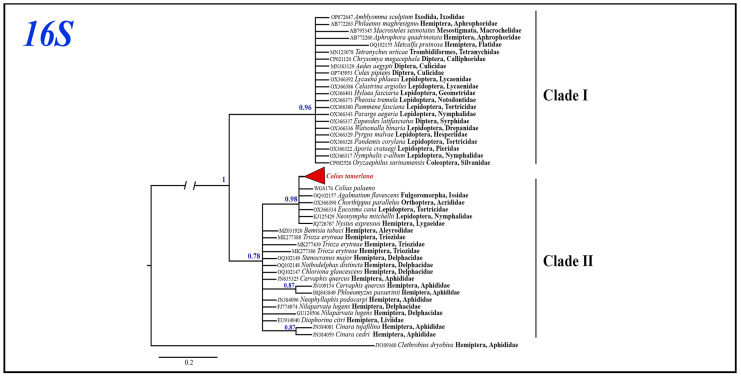
The Bayesian tree of *16S* gene fragment inferred from 57 *Wolbachia* samples. Numbers at nodes indicate Bayesian posterior probabilities.

Bayesian analysis of the *Wolbachia 16S* gene fragment based on 43 most similar sequences with known host species mined from GenBank recovered two clades with high (clade I, BS = 0.96) and low (clade II, BS = 0.78) support (Figure 6). All *Wolbachia 16S* sequences isolated from infected *C. mongola*/*C. tamerlana* specimens were grouped within clade II together with the *Wolbachia* alleles found in three insect orders: Lepidoptera, Hemiptera and Orthoptera. The clade I included *Wolbachia* alleles found in various Lepidoptera families, Diptera, Coleoptera, Hemiptera and mites (Parasitiformes, Trombidiformes). Maximum p-distances within clade I and clade II were as high as 1.5% and 0.8%, respectively; maximum p-distances between the two clades were 1.8%.

**Figure 7:**
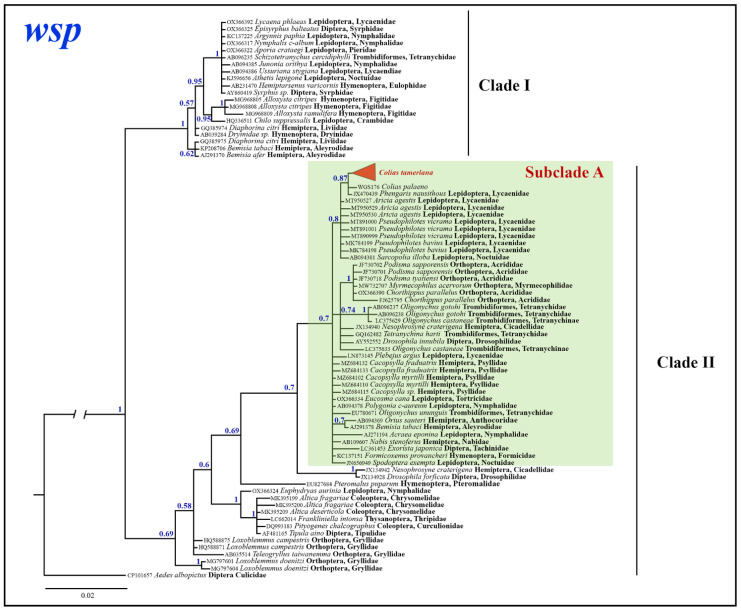
The Bayesian tree of *wsp* gene fragment inferred from 88 *Wolbachia* samples. Numbers at nodes indicate Bayesian posterior probabilities.

Bayesian analysis of the *Wolbachia wsp* gene fragment based on 74 most similar sequences with known host species mined from GenBank revealed two clades: well-supported clade I (BS = 1) and weakly supported clade II (BS = 0.69). The latter, in turn, is subdivided into several unsupported lineages (Figure 7). Obtained in the present study *Wolbachia wsp* sequences from *C. mongola*/*C. tamerlana* clustered within the subclade A of the *wsp* clade II, which unites *Wolbachia wsp* alleles isolated from members of the insect orders Lepidoptera, Orthoptera, Hemiptera, Diptera, Hymenoptera and spider mites (Trombidiformes). Maximum p-distances within *wsp* clade I and *wsp* clade II were as high as 1.8% and 5.7%, respectively; between the two clades was 7.1%; and within subclade A was 3%.

**Figure 8:**
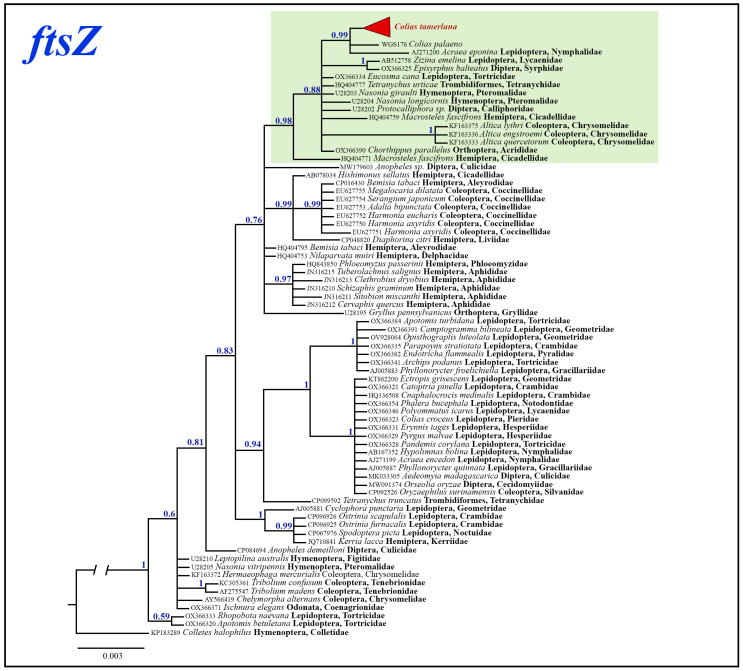
The Bayesian tree of *ftsZ* gene fragment inferred from 85 *Wolbachia* samples. Numbers at nodes indicate Bayesian posterior probabilities.

For the phylogenetic analysis of the *Wolbachia ftsZ* gene fragment, we used additional 71 of the most similar sequences with known host species obtained from GenBank. Bayesian analysis revealed several highly supported clades (Bayesian posterior probability > 0.95); however, deeper nodes mostly remained poorly resolved (Figure 8). *Wolbachia* samples isolated from *C. tamerlana* specimens were grouped within a well-supported clade (BS = 0.98), together with members of insect orders Lepidoptera, Orthoptera, Hemiptera, Diptera, Hymenoptera, Coleoptera and Trombidiformes mites. Maximum p-distances within this clade were as high as 1.77%; maximum p-distances among all analyzed *ftsZ* samples reached 2.37%.

## 4. Discussion

### 4.1. Molecular Analysis and Taxonomy of C. mongola/C. tamerlana

Mitochondrial DNA is widely used in phylogenetic reconstructions, taxonomic studies and species identification and delimitation due to distinct advantages over other molecular markers [76,77]. However, numerous studies underline the limited application of DNA barcoding in the context of incomplete lineage sorting, mitochondrial introgression and infection by endosymbionts, such as *Wolbachia* [78,79,80,81,82,83,84]. Our analysis of the mitochondrial barcodes alone revealed four differentiated clades within *C. mongola*/*C. tamerlana* with genetic distances reaching 2.89%. Such values are comparable to “standard” species-level *COI* divergence empirically estimated for Lepidoptera [85,86,87,88] and suggest a relatively old separation of the recovered *COI* lineages. In fact, if we relied solely on *COI* barcodes, the possible conclusion would have been that the observed lineages might represent unique cryptic species, especially taking into account the comparatively young radiation of the genus *Colias* [43,44]. However, this conclusion was not supported by subsequent analyses of the nuclear genes and phenotypic traits. The recovered mtDNA phylogenetic structure was not corroborated by the nuclear data: the specimens bearing different *COI* haplotypes were randomly distributed across the nucDNA phylogenetic tree. The nuclear sequences showed no signs of significant divergence between the clades, delimited by mitochondrial barcodes, forming a single, nearly unstructured entity. Furthermore, butterflies collected from the remote geographical localities show no traces of morphological differentiation despite their phenotypic variability. The only exception is *C. tamerlana sidonia*, the geographically isolated taxon from the Lake Khovsgol area (Mongolia), which differs from the typical *C. mongola*/*C. tamerlana* in its larger size, less developed dark suffusion on the fore- and hindwings and large submarginal light spots. Such prominent morphological differences even force some authors to consider *sidonia* as a subspecies of the more easterly distributed taxon, *C. nastes*. However, our data unequivocally show that *sidonia* undoubtedly belongs to the *C. mongola*/*C. tamerlana* complex, sharing common and geographically widespread *COI* haplotype hb1 with the latter. It should also be noted that in the case of cryptic species, we should expect geographic isolation (in case of allopatry) or niche separation (in case of sympatry) of revealed mtDNA clusters, which we consider as a putative cryptic species [89,90,91]. Certainly, this is not the case for *C. mongola*/*C. tamerlana*, where butterflies of different *COI* haplogroups were found flying together syntopically (in the same habitat) and synchronously (at the same time), in other words in complete sympatry without any niche separation.

The taxonomic status of *C. mongola* and *C. tamerlana* and the relationships of this pair of taxa and allied species is also a subject of longstanding debates. Analysis of the century-old type specimens, namely the lectotype of *C. tamerlana*, the paralectotype of *C. tamerlana* and the lectotype of *C. mongola*, along with *C. tamerlana sidonia* specimens from the type locality, presumably belonging to the type series, allowed us to shed light on this very controversial issue of the *Colias* taxonomy. The type locality of *C. mongola* has been contradictorily interpreted in the taxonomic literature and cannot be clearly ascertained. Thus, the limited number of specimens originating from the type series stored in the museum collections is the only reliable source of molecular data. Here, using NGS approach, we recover and analyze DNA barcodes of these old museum specimens. We confirm the conspecificity of *C. tamerlana* and *C. mongola* and show that the type specimens of these taxa share the same *COI* haplotype hb1, common and widely spread over a large geographical area from northwestern China through the south Siberian part of Russia to western Mongolia.

### 4.2. Wolbachia Infection in C. mongola/C. tamerlana

Recent investigations have suggested that *Wolbachia* infection is common and widespread in Lepidoptera [92,93,94]; however, large-scale and comprehensive studies devoted to the incidence, pattern of *Wolbachia* infection and its impact on phylogenetic inferences of host species are still scarce [95,96,97,98,99,100,101,102,103,104]. To date, *Wolbachia* infection has been reported only for a few *Colias* species, namely *C. palaeno*, *C. hyale* (Linnaeus, 1758), *C. poliographus* Motschulsky, 1861 and *C. croceus* [46,94,105,106]. Here, we designated a new *Wolbachia* allele *w*Tam, which had not been previously recorded in *Colias*. Surprisingly, none of the available *Wolbachia wsp* and *ftsZ* STs (sequence types) found in *Colias* and deposited in public databases GenBank (https://blast.ncbi.nlm.nih.gov, accessed on 20 July 2023) and PubMLST-*Wolbachia* (https://pubmlst.org/organisms/wolbachia-spp, accessed on 25 July 2023) fall within the sequences with the highest percentage identity much to *w*Tam. However, our personal unpublished data suggest that alleles similar to *w*Tam can be found in other *Colias* taxa.

The *Wolbachia* allele found in *C. mongola*/*C. tamerlana* is shared among different insect species, families and even orders. *Wolbachia* infection is mainly vertically transmitted to the progeny via maternal cytoplasm. Accordingly, one should expect the phylogeny of *Wolbachia* to be consistent with the phylogeny of their hosts. Notwithstanding, the horizontal transfer of *Wolbachia* between insect hosts have been suggested for many insect taxa [107,108,109,110,111,112]; however, the mechanisms of this phenomenon remain to be characterized. It has been suggested that *Wolbachia* can shift between distantly related hosts through host-parasitoid interactions [113,114,115,116], shared host plants [117,118,119,120], hybridisation events [99,121,122,123,124] and predator-prey associations [125].

*Wolbachia 16S* sequence obtained in our study is shared by two Lepidoptera species, nymphalid *Neonympha mitchellii* French, 1889 distributed in the eastern USA and tortricid moth *Eucosma cana* (Haworth, 1811), the meadow grasshopper *Chorthippus parallelus* (Zetterstedt, 1821) (Orthopthera, Acrididae), the false chinch bug *Nysius expressus* Distant, 1883 (Hemiptera, Lygaeidae) and the planthopper *Agalmatium flavescens* (Olivier, 1791) (Hemiptera, Issidae). The *Wolbachia wsp* sequence is shared by the lycaenid butterfly *Phengaris nausithous* (Lepidoptera, Lycaenidae). Our data evidenced that the same *Wolbachia* strain may occur in very distant, not closely related taxa. Thus, we confirm previous studies suggesting that horizontal transmissions events are quite common in nature [126,127]. Interestingly, the very similar *wsp* and *ftsZ* STs to *C. mongola*/*C. tamerlana* carry spider mites (Acari, Trombidiformes). Mites are known as common parasites for Lepidoptera and other insects and arthropods and have been suggested as potential vectors for *Wolbachia* transmission [128,129]. Occurrence of closely related *Wolbachia* strains in butterflies and Trombidiformes indicate that the *Wolbachia* host switches in Lepidoptera might be caused by mites.

Screening for *Wolbachia* revealed infection in 14 specimens (out of 88 tested), suggesting a relatively low infection rate in *C. mongola*/*C. tamerlana* (prevalence: 16%). One of the most intriguing results obtained in the present study is that all infected specimens were females of certain haplotype (hp2), indicating possible sex and haplotype selectiveness of the *Wolbachia* infection. However, detailed studies based on more extensive sampling and thorough inspection for *Wolbachia* in both reproductive and somatic tissues are needed in order to confirm this hypothesis. To our knowledge, such sex-biased, selective and total infection of certain mitochondrial lineage have never been observed in Lepidoptera. Moreover, our personal unpublished data suggest that such patterns of selective infection can be found in other *Colias* taxa, presumably being a general characteristic of the genus. It also should be noted that a surprisingly low number of publications devoted to analysis of *Wolbachia* infection in Lepidoptera consider *Wolbachia* prevalence in males and females independently [130,131], while the sex-dependent impact of *Wolbachia* on its hosts is highly expected.

Interestingly, a somewhat similar pattern has been observed in another pierid genus *Eurema* (Lepidoptera, Pieridae), belonging to the same subfamily Coliadinae, where the sex-biased female lineages of *Wolbachia* were discovered in two Japanese species [132,133,134]. The *Wolbachia* allele *w*Fem was found at low frequencies only in the *Eurema* females and has not been observed in the males. A causative role of *w*Fem allele in feminization, a well-known manipulation effect deployed by *Wolbachia* [135], has been proposed. It has been shown that antibiotic treatment of infected larvae leads to occurrence of intersex individuals, while treatment of adult females results in all-male progeny. We cannot exclude that the sex-biased *Wolbachia* infection observed in *C. mongola*/*C. tamerlana* is also a consequence of feminization, a phenomenon that has been rarely encountered within Lepidoptera [136]. However, further comprehensive studies based on the lab experiments and analysis of additional material are needed to confirm such assumptions.

## 5. Conclusions

Our study is the first large-scale investigation aimed at a detailed analysis of phylogeographical structure, geographical distribution and taxonomy of two enigmatic *Colias* taxa with controversial taxonomic status, *C*. *mongola* and *C. tamerlana.* Our analysis clustered DNA barcodes that were obtained for the present study in four distinct haplogroups; however, no association between nuclear genes and mitochondrial clusters, as well as between the distribution of mitochondrial haplotypes and geography, has been revealed. Using NGS approach, we recover and analyze DNA barcodes of century-old *C*. *mongola* and *C*. *tamerlana* type specimens. We show that the type specimens of these taxa share the same *COI* haplotype. These results confirm the conspecificity of *C. tamerlana* and *C. mongola*, solving a longstanding question about their taxonomic status, also demonstrating that the application of modern techniques is of a great importance in cases when the type locality is unknown or cannot be clearly ascertained, and old material originated from the type series is the only reliable source of molecular data. We analyze the presence and prevalence of *Wolbachia* in *C. mongola*/*C. tamerlana* and found strong correlations between sex, specific mitochondrial lineage and *Wolbachia* infection. Phylogenetic analysis placed the *Wolbachia* strain of *C. mongola*/*C. tamerlana* together with members of different insect families and even orders, indicating multiple events of host shifts, thus being consistent with the former studies, evidencing that horizontal transmission is a common mechanism of *Wolbachia* expansion. We conclude that the occurrence of deep intraspecific divergences of DNA barcodes is not necessarily a consequence of cryptic speciation but instead can be a result of *Wolbachia* infection and some other, most likely, environmental factors.

## 6. Taxonomic Conclusions

The nomenclature of *C. tamerlana* and *C. mongola* is a subject of a longstanding debate [1,3,13]. Here we follow the logic of Grieshuber [1], who pointed out that under the International Code of Zoological Nomenclature (Article 8.1.2.) a work is regarded as having been published only when it becomes available. The earliest confirmed date for Romanoff`s book is 18.12.1897, when the copy of 9th volume has been received by the Harvard University library [1]. Consequently, the name *tamerlana* has priority over *mongola.* Taking into account the molecular data obtained in the present study, we confirm the conspecificity of the taxa in question and consider *Colias nastes mongola* Alphéraky, 1897 a junior subjective synonym of *Colias tamerlana* Staudinger, 1897, **syn. nov**. We did not find any molecular evidence supporting subspecies status of *Colias mongola ukokana* Korb and Yakovlev, 2000 and *Colias cocandica sidonia* Weiss, 1968, despite pronounced differences in the external morphological characters of the latter. These populations do not show any signs of divergence and should be considered as the nominotypical *Colias tamerlana*.

Thus, we suggest the following rearrangements:

*Colias nastes mongola* Alphéraky, 1897 = *Colias tamerlana* Staudinger, 1897, **syn. nov**.
*Colias mongola ukokana* Korb and Yakovlev, 2000 = *Colias tamerlana* Staudinger, 1897, **syn. nov**.
*Colias cocandica sidonia* Weiss, 1968 = *Colias tamerlana* Staudinger, 1897, **syn. nov**.

## Figures and Tables

**Table 1:** Summary of mitochondrial haplotype diversity of *C. mongola/C. tamerlana.* The number of individuals sequenced (*N*), the number of haplogroups (*HG*) and haplotypes (*H*) revealed, the number of polymorphic sites (*S*), nucleotide (π) and haplotype (*h*) diversities are given. Tajima’s D, Fu and Li’s D, Fu and Li’s F, Fu’s Fs, max. p-distance, within and between group divergence values with standard deviation (in brackets) are shown.

*N*	*HG*	*H*	*S*	π	*h*	Tajima’s D	Fu and Li’s D	Fu and Li’s F	Fu’s Fs	Max. P-Distance	Within Group Divergence	Between Group Divergence
101	4	12	28	0.0138656	0.7874	2.01246	1.21273	1.82127	7.422	2.89%	HP_I 0.04% (± 0.02%)	HP_I/HP_II 2.56% (±0.59%)
						0.1 > *p* > 0.05	*p* > 0.1	*p* < 0.05	*p* = 0.001	(±0.62%)	HP_II 0.00% (±0.00%)	HP_I/HP_III 2.40% (±0.57%)
											HP_III 0.07% (±0.05%)	HP_I/HP_IV 2.56% (±0.58%)
											HP_I 0.04% (±0.03%)	HP_II/HP_III 1.39% (±0.42%)
												HP_II/HP_IV 1.23% (±0.41%)
												HP_III/HP_IV 1.41% (±0.43%)

**Table 2:** Haplogroup and haplotype composition (the number of individuals constituted a haplogroup/haplotype (*n*), and the number of sampling sites where haplogroup/haplotype was found (*Sn*) are given) and its geographical distribution (the number of individuals (*n*), the number of haplogroups (*HGn*), the number of haplotypes (*Hn*) found in each locality and haplotype constitution are shown). *—sampling sites correspond to Figure 3b.

Haplogroup	*n*	*Sn*	*COI* Haplotype	*n*	*Sn*	Sampling Site *	*n*	*HGn*	*Hn*	*COI* Haplotype (*n*)
HP_I	26	9	hp1a	5	4	01	7	2	2	hp1b (2), hp3a (5)
			hp1b	20	6	02	3	2	3	hp1a (1), hp1c (1), hp3b (1)
			hp1c	1	1	03	3	1	1	hp2 (3)
HP_II	17	10	hp2	17	10	04	1	1	1	hp2 (1)
HP_III	42	11	hp3a	35	9	05	5	3	3	hp2 (2), hp3a (2), hp4a (1)
			hp3b	1	1	06	6	3	3	hp2 (2), hp3c (1), hp4a (3)
			hp3c	1	1	07	2	1	1	hp3a (2)
			hp3d	3	1	08	1	1	1	hp4c (1)
			hp3e	2	1	09	35	4	7	hp1a (2), hp2 (1), hp3a (17), hp3d (3), hp3e (2), hp4a (9), hp4b (1)
HP_IV	16	4	hp4a	14	4	10	5	3	3	hp1a (1), hp2 (1), hp3a (3)
			hp4b	1	1	11	2	1	1	hp3a (2)
			hp4c	1	1	12	2	1	1	hp3a (2)
						13	2	2	2	hp2 (1), hp3a (1)
						14	9	3	4	hp1a (2), hp1b (5), hp2 (1), hp4a (1)
						15	2	1	1	hp1b (2)
						16	1	1	1	hp2 (1)
						17	10	3	3	hp1b (5), hp2 (4), hp3a (1)
						18	4	1	1	hp1b (4)

## Data Availability

All the analyzed DNA sequences are available via the GenBank links provided.

## Supplementary Materials

The following supporting information can be downloaded at: https://www.mdpi.com/article/10.3390/insects14120943/s1, Table S1: List of the studied specimens. Table S2: GenBank accession numbers for sequence records. Table S3: A detailed summary of the nucleotide variability of the studied *Ca-ATPase*, *CAD* and *H3* nuclear genes fragments among the sequenced samples of *C.mongola*/*C. tamerlana*.
