# Supplementary material for: Phylogeography of Two Enigmatic Sulphur Butterflies, Colias mongola Alphéraky, 1897 and Colias tamerlana Staudinger, 1897 (Lepidoptera, Pieridae), with Relations to Wolbachia Infection
Source: Insects. 2023 Dec 13;14(12):943. doi: 10.3390/insects14120943 (PMC10743618; doi:10.3390/insects14120943)
Supplement: Supplementary file 1 [file insects-14-00943-s001.zip › Table S1.pdf]

Table S1. List of the studied specimens. LT – lectotype; PT – paratype; PLT – paralectotype; *— DNA barcodes, mined from BOLD; **—numbers in brackets correspond to sampling localities, shown in Figure 3b. Attribution of samples to *mongola*/*tamerlana* was based on a geographical distribution of the corresponding taxa suggested in previous studies.

Taxon	Sample ID	Sex	*COI* haplotype	*Wolbachia*			Year	Locality
				16S	*wsp*	*ftsZ*		
tamerlana	Akr01	♂	hp3a	-	-	-	2016	(01)** China, Xinjiang
tamerlana	Akr02	♂	hp3a	-	-	-	2016	(01) China, Xinjiang
tamerlana	Akr03	♀	hp3a	-	-	-	2016	(01) China, Xinjiang
tamerlana	Akr04	♀	hp3a	-	-	-	2016	(01) China, Xinjiang
tamerlana	CL162	♂	hp3a	-	-	-	2016	(01) China, Xinjiang
tamerlana LT	21128B07	♂	hp1b	n/a	n/a	n/a	1896	(01) China, Xinjiang
tamerlana PLT	20022A02	♀	hp1b	n/a	n/a	n/a	1896	(01) China, Xinjiang
mongola	Mnt03	♂	hp1b	-	-	-	2018	(02) Mongolia, Hovd
mongola	Mnt04	♀	hp3b	-	-	-	2018	(02) Mongolia, Hovd
mongola	CL34m	♂	hp1c	-	-	-	2015	(02) Mongolia, Hovd
mongola	Mnt54	♀	hp1b	-	-	-	2003	(17) Mongolia, Hovd
mongola	Mnt55	♀	hp2	+	+	+	2003	(17) Mongolia, Hovd
mongola	Mnt56	♀	hp1b	-	-	-	2003	(17) Mongolia, Hovd
mongola	Mnt57	♀	hp1b	-	-	-	2003	(17) Mongolia, Hovd
mongola	Mnt58	♀	hp2	+	+	+	2003	(17) Mongolia, Hovd
mongola	Mnt59	♀	hp3a	-	-	-	2003	(17) Mongolia, Hovd
mongola	Mnt60	♀	hp2	+	+	+	2003	(17) Mongolia, Hovd
mongola	Mnt61	♂	hp1b	-	-	-	2003	(17) Mongolia, Hovd
mongola	Mnt62	♂	hp2	+	+	+	2003	(17) Mongolia, Hovd
mongola	Mnt63	♂	hp1b	-	-	-	2003	(17) Mongolia, Hovd
mongola	Mnt01	♀	hp2	+	+	+	2013	(03) Mongolia, Gobi-Altai
mongola	Mnt02	♀	hp2	+	+	+	2013	(03) Mongolia, Gobi-Altai
mongola	CL41m	♀	hp2	+	+	+	2017	(03) Mongolia, Gobi-Altai
mongola	Nsk16	♂	hp2	-	-	-	2005	(04) Mongolia, Bayan-Ulegej
mongola	CL39m	♀	hp2	+	+	+	2017	(05) Mongolia, Bayan-Ulegej
mongola	CL40m	♀	hp3a	-	-	-	2017	(05) Mongolia, Bayan-Ulegej
mongola	CL43m	♀	hp2	+	+	+	2017	(05) Mongolia, Bayan-Ulegej
mongola	Mnt24z	♀	hp3a	-	-	-	2017	(05) Mongolia, Bayan-Ulegej
mongola	Mnt25z	♀	hp4a	-	-	-	2017	(05) Mongolia, Bayan-Ulegej
mongola	Mnt53	♀	hp2	+	+	+	2004	(16) Mongolia, Zavkhan
mongola ukokana	Mnt18	♀	hp2	+	+	+	2022	(06) Russia, Republic of Altai
mongola ukokana	Mnt19	♀	hp3c	-	-	-	2022	(06) Russia, Republic of Altai
mongola ukokana	Mnt20	♂	hp4a	-	-	-	2022	(06) Russia, Republic of Altai
mongola ukokana	Mnt21	♂	hp4a	-	-	-	2022	(06) Russia, Republic of Altai
mongola ukokana	Mnt22	♂	hp4a	-	-	-	2022	(06) Russia, Republic of Altai
mongola ukokana	Mnt23	♂	hp2	-	-	-	2022	(06) Russia, Republic of Altai
mongola ukokana	Nsk014	♂	hp3a	n/a	n/a	n/a	1997	(07) Russia, Republic of Altai
mongola ukokana	Mnt34z	♂	hp3a	n/a	n/a	n/a	1997	(07) Russia, Republic of Altai
mongola ukokana PT	Nsk015	♂	hp4c	n/a	n/a	n/a	1995	(08) Russia, Republic of Altai
mongola ukokana	Mnt24	♂	hp3a	-	-	-	2001	(09) Russia, Republic of Altai
mongola ukokana	Mnt25	♀	hp1a	-	-	-	2001	(09) Russia, Republic of Altai
mongola ukokana	Mnt26	♂	hp4a	-	-	-	2001	(09) Russia, Republic of Altai
mongola ukokana	Mnt27	♀	hp3a	-	-	-	2001	(09) Russia, Republic of Altai
mongola ukokana	Mnt28	♀	hp4a	-	-	-	2001	(09) Russia, Republic of Altai
mongola ukokana	Mnt29	♂	hp4a	-	-	-	2001	(09) Russia, Republic of Altai

mongola ukokana	Mnt30	♂	hp3a	-	-	-	2001	(09) Russia, Republic of Altai
mongola ukokana	Mnt31	♂	hp3a	-	-	-	2001	(09) Russia, Republic of Altai
mongola ukokana	Mnt32	♂	hp4a	-	-	-	2001	(09) Russia, Republic of Altai
mongola ukokana	Mnt33	♂	hp3a	-	-	-	2001	(09) Russia, Republic of Altai
mongola ukokana	Mnt34	♂	hp3a	-	-	-	2001	(09) Russia, Republic of Altai
mongola ukokana	Mnt35	♂	hp4a	-	-	-	2001	(09) Russia, Republic of Altai
mongola ukokana	Mnt36	♂	hp3a	-	-	-	2001	(09) Russia, Republic of Altai
mongola ukokana	Mnt37	♂	hp3a	-	-	-	2001	(09) Russia, Republic of Altai
mongola ukokana	Mnt38	♂	hp3a	-	-	-	2001	(09) Russia, Republic of Altai
mongola ukokana	Mnt39	♂	hp3a	-	-	-	2001	(09) Russia, Republic of Altai
mongola ukokana	Mnt44	♂	hp4a	-	-	-	2001	(09) Russia, Republic of Altai
mongola ukokana	Mnt45	♂	hp4a	-	-	-	2001	(09) Russia, Republic of Altai
mongola ukokana	Mnt46	♂	hp3a	-	-	-	2001	(09) Russia, Republic of Altai
mongola ukokana	Mnt47	♂	hp3d	-	-	-	2001	(09) Russia, Republic of Altai
mongola ukokana	Mnt48	♂	hp3a	-	-	-	2001	(09) Russia, Republic of Altai
mongola ukokana	Mnt49	♂	hp3a	-	-	-	2001	(09) Russia, Republic of Altai
mongola ukokana	Mnt50	♂	hp3d	-	-	-	2001	(09) Russia, Republic of Altai
mongola ukokana	Mnt51	♂	hp3a	-	-	-	2001	(09) Russia, Republic of Altai
mongola ukokana	Ku01	♀	hp3e	-	-	-	2022	(09) Russia, Republic of Altai
mongola ukokana	Ku02	♀	hp2	+	+	+	2022	(09) Russia, Republic of Altai
mongola ukokana	Ku03	♂	hp4a	-	-	-	2022	(09) Russia, Republic of Altai
mongola ukokana	Ku04	♂	hp4a	-	-	-	2022	(09) Russia, Republic of Altai
mongola ukokana	Mnt28z	♂	hp3a	-	-	-	2022	(09) Russia, Republic of Altai
mongola ukokana	Mnt29z	♀	hp3e	-	-	-	2022	(09) Russia, Republic of Altai
mongola ukokana	Mnt30z	♂	hp3d	-	-	-	2022	(09) Russia, Republic of Altai
mongola ukokana	LOWA141-06*	♂	hp3a	n/a	n/a	n/a	1999	(09) Russia, Republic of Altai
mongola ukokana	LOWA818-06*	♂	hp3a	n/a	n/a	n/a	1999	(09) Russia, Republic of Altai
mongola ukokana	LOWA142-06*	♂	hp4b	n/a	n/a	n/a	1999	(09) Russia, Republic of Altai
mongola ukokana	CL42m	♀	hp1a	-	-	-	2017	(09) Russia, Republic of Altai
mongola ukokana	Mnt17	♀	hp1a	-	-	-	2022	(10) Russia, Republic of Altai
mongola ukokana	Mnt40	♀	hp2	+	+	+	2003	(10) Russia, Republic of Altai
mongola ukokana	Mnt41	♂	hp3a	-	-	-	2003	(10) Russia, Republic of Altai
mongola ukokana	Mnt42	♂	hp3a	-	-	-	2003	(10) Russia, Republic of Altai
mongola ukokana	Mnt43	♂	hp3a	-	-	-	2003	(10) Russia, Republic of Altai
mongola ukokana	Mnt09	♂	hp3a	-	-	-	2002	(11) Russia, Republic of Altai
mongola ukokana	Mnt10	♂	hp3a	-	-	-	2002	(11) Russia, Republic of Altai
mongola ukokana	Mnt08	♂	hp3a	-	-	-	2003	(12) Russia, Republic of Altai
mongola ukokana	Mnt12	♂	hp3a	-	-	-	2003	(12) Russia, Republic of Altai
mongola	Mnt14	♀	hp2	+	+	+	2010	(13) Russia, Republic of Tyva
mongola	Mnt15	♀	hp3a	-	-	-	2010	(13) Russia, Republic of Tyva
mongola	Mnt07	♂	hp2	-	-	-	2002	(14) Russia, Republic of Tyva
mongola	CL35m	♀	hp1b	-	-	-	2002	(14) Russia, Republic of Tyva
mongola	CL37m	♂	hp1b	-	-	-	2002	(14) Russia, Republic of Tyva
mongola	CL38m	♀	hp1a	-	-	-	2002	(14) Russia, Republic of Tyva
mongola	CL54m	♂	hp1b	-	-	-	2002	(14) Russia, Republic of Tyva
mongola	CL55m	♂	hp4a	-	-	-	2002	(14) Russia, Republic of Tyva
mongola	CL56m	♂	hp1b	-	-	-	2002	(14) Russia, Republic of Tyva
mongola	CL57m	♀	hp1b	-	-	-	2002	(14) Russia, Republic of Tyva
mongola	Mnt26z	♂	hp1a	-	-	-	2002	(14) Russia, Republic of Tyva
mongola	Mnt05	♀	hp1b	-	-	-	2001	(15) Russia, Republic of Tyva
mongola	Mnt06	♀	hp1b	-	-	-	2001	(15) Russia, Republic of Tyva
mongola LT	20022A03	♂	hp1b	n/a	n/a	n/a	?	(??) see text for explanation

sidonia	S1-22064B02	♀	hp1b	n/a	n/a	n/a	1967	(18) Mongolia, Khovsgol
sidonia	S2-22064B03	♀	hp1b	n/a	n/a	n/a	1966	(18) Mongolia, Khovsgol
sidonia	S3-22064B04	♂	hp1b	n/a	n/a	n/a	1967	(18) Mongolia, Khovsgol
sidonia	S4-22064B05	♂	hp1b	n/a	n/a	n/a	1967	(18) Mongolia, Khovsgol
