# Supplementary material for: Phylogeography of Two Enigmatic Sulphur Butterflies, Colias mongola Alphéraky, 1897 and Colias tamerlana Staudinger, 1897 (Lepidoptera, Pieridae), with Relations to Wolbachia Infection
Source: Insects. 2023 Dec 13;14(12):943. doi: 10.3390/insects14120943 (PMC10743618; doi:10.3390/insects14120943)
Supplement: Supplementary file 1 [file insects-14-00943-s001.zip › Table_S2.pdf]

Table S2. List of studied materials with GenBank accession numbers for sequence records.

Taxon	Sample ID	*COI* haplotype	GenBank Accession Number				*Wolbachia*		
			COI	*CAD*	*H3*	*CA-ATPase*	*16S*	*wsp*	*fts-z*
tamerlana	Akr01	hp3a	OP946559	-	-	-	-	-	-
tamerlana	Akr02	hp3a	OP946560	-	-	-	-	-	-
tamerlana	Akr03	hp3a	OP946561	-	-	-	-	-	-
tamerlana	Akr04	hp3a	OP946562	-	-	-	-	-	-
tamerlana	CL162	hp3a	OP946563	-	-	-	-	-	-
tamerlana LT	21128B07	hp1b	OP946564	-	-	-	-	-	-
tamerlana PLT	20022A02	hp1b	OP946565	-	-	-	-	-	-
mongola	Mnt03	hp1b	OP946566	-	-	-	-	-	-
mongola	Mnt04	hp3b	OP946567	OQ192178	OQ192144	OQ192161	-	-	-
mongola	CL34m	hp1c	OP946568	OQ192179	OQ192145	OQ192162	-	-	-
mongola	Mnt54	hp1b	OP946569	-	-	-	-	-	-
mongola	Mnt55	hp2	OP946570	-	-	-	OQ155222	OQ192116	OQ192130
mongola	Mnt56	hp1b	OP946571	-	-	-	-	-	-
mongola	Mnt57	hp1b	OP946572	-	-	-	-	-	-
mongola	Mnt58	hp2	OP946573	-	-	-	OQ155223	OQ192117	OQ192131
mongola	Mnt59	hp3a	OP946574	-	-	-	-	-	-
mongola	Mnt60	hp2	OP946575	-	-	-	OQ155224	OQ192118	OQ192132
mongola	Mnt61	hp1b	OP946576	-	-	-	-	-	-
mongola	Mnt62	hp2	OP946577	-	-	-	OQ155225	OQ192119	OQ192133
mongola	Mnt63	hp1b	OP946578	-	-	-	-	-	-
mongola	Mnt01	hp2	OP946579	-	-	-	OQ155226	OQ192120	OQ192134
mongola	Mnt02	hp2	OP946580	-	-	-	OQ155227	OQ192121	OQ192135
mongola	CL41m	hp2	OP946581	-	-	-	OQ155228	OQ192122	OQ192136
mongola	Nsk16	hp2	OP946582	-	-	-	-	-	-
mongola	CL39m	hp2	OP946583	OQ192180	OQ192146	OQ192163	OQ155229	OQ192123	OQ192137
mongola	CL40m	hp3a	OP946584	OQ192181	OQ192147	OQ192164	-	-	-
mongola	CL43m	hp2	OP946585	OQ192182	OQ192148	OQ192165	OQ155230	OQ192124	OQ192138
mongola	Mnt24z	hp3a	OP946586	-	-	-	-	-	-
mongola	Mnt25z	hp4a	OP946587	-	-	-	-	-	-

mongola	Mnt53	hp2	OP946588	-	-	-	OQ155231	OQ192125	OQ192139
mongola ukokana	Mnt18	hp2	OP946589	OQ192183	OQ192149	OQ192166	OQ155232	OQ192126	OQ192140
mongola ukokana	Mnt19	hp3c	OP946590	-	-	-	-	-	-
mongola ukokana	Mnt20	hp4a	OP946591	OQ192184	OQ192150	OQ192167	-	-	-
mongola ukokana	Mnt21	hp4a	OP946592	OQ192185	OQ192151	OQ192168	-	-	-
mongola ukokana	Mnt22	hp4a	OP946593	-	-	-	-	-	-
mongola ukokana	Mnt23	hp2	OP946594	-	-	-	-	-	-
mongola ukokana	Nsk014	hp3a	OP946595	-	-	-	-	-	-
mongola ukokana	Mnt34z	hp3a	OP946596	-	-	-	-	-	-
mongola ukokana PT	Nsk015	hp4c	OP946597	-	-	-	-	-	-
mongola ukokana	Mnt24	hp3a	OP946598	-	-	-	-	-	-
mongola ukokana	Mnt25	hp1a	OP946599	-	-	-	-	-	-
mongola ukokana	Mnt26	hp4a	OP946600	-	-	-	-	-	-
mongola ukokana	Mnt27	hp3a	OP946601	-	-	-	-	-	-
mongola ukokana	Mnt28	hp4a	OP946602	-	-	-	-	-	-
mongola ukokana	Mnt29	hp4a	OP946603	-	-	-	-	-	-
mongola ukokana	Mnt30	hp3a	OP946604	-	-	-	-	-	-
mongola ukokana	Mnt31	hp3a	OP946605	-	-	-	-	-	-
mongola ukokana	Mnt32	hp4a	OP946606	-	-	-	-	-	-
mongola ukokana	Mnt33	hp3a	OP946607	-	-	-	-	-	-
mongola ukokana	Mnt34	hp3a	OP946608	-	-	-	-	-	-
mongola ukokana	Mnt35	hp4a	OP946609	-	-	-	-	-	-
mongola ukokana	Mnt36	hp3a	OP946610	-	-	-	-	-	-
mongola ukokana	Mnt37	hp3a	OP946611	-	-	-	-	-	-
mongola ukokana	Mnt38	hp3a	OP946612	-	-	-	-	-	-
mongola ukokana	Mnt39	hp3a	OP946613	-	-	-	-	-	-
mongola ukokana	Mnt44	hp4a	OP946614	-	-	-	-	-	-
mongola ukokana	Mnt45	hp4a	OP946615	-	-	-	-	-	-
mongola ukokana	Mnt46	hp3a	OP946616	-	-	-	-	-	-
mongola ukokana	Mnt47	hp3d	OP946617	-	-	-	-	-	-
mongola ukokana	Mnt48	hp3a	OP946618	-	-	-	-	-	-
mongola ukokana	Mnt49	hp3a	OP946619	-	-	-	-	-	-
mongola ukokana	Mnt50	hp3d	OP946620	-	-	-	-	-	-

mongola ukokana	Mnt51	hp3a	OP946621	-	-	-	-	-	-
mongola ukokana	Ku01	hp3e	OP946622	OQ192186	OQ192152	OQ192169	-	-	-
mongola ukokana	Ku02	hp2	OP946623	OQ192187	OQ192153	OQ192170	OQ155233	OQ192127	OQ192141
mongola ukokana	Ku03	hp4a	OP946624	OQ192188	OQ192154	OQ192171	-	-	-
mongola ukokana	Ku04	hp4a	OP946625	OQ192189	OQ192155	OQ192172	-	-	-
mongola ukokana	Mnt28z	hp3a	OP946626	OQ192190	OQ192156	OQ192173	-	-	-
mongola ukokana	Mnt29z	hp3e	OP946627	OQ192191	OQ192157	OQ192174	-	-	-
mongola ukokana	Mnt30z	hp3d	OP946628	OQ192192	OQ192158	OQ192175	-	-	-
mongola ukokana	CL42m	hp1a	OP946629	OQ192193	OQ192159	OQ192176	-	-	-
mongola ukokana	Mnt17	hp1a	OP946630	OQ192194	OQ192160	OQ192177	-	-	-
mongola ukokana	Mnt40	hp2	OP946631	-	-	-	OQ155234	OQ192128	OQ192142
mongola ukokana	Mnt41	hp3a	OP946632	-	-	-	-	-	-
mongola ukokana	Mnt42	hp3a	OP946633	-	-	-	-	-	-
mongola ukokana	Mnt43	hp3a	OP946634	-	-	-	-	-	-
mongola ukokana	Mnt09	hp3a	OP946635	-	-	-	-	-	-
mongola ukokana	Mnt10	hp3a	OP946636	-	-	-	-	-	-
mongola ukokana	Mnt08	hp3a	OP946637	-	-	-	-	-	-
mongola ukokana	Mnt12	hp3a	OP946638	-	-	-	-	-	-
mongola	Mnt14	hp2	OP946639	-	-	-	OQ155235	OQ192129	OQ192143
mongola	Mnt15	hp3a	OP946640	-	-	-	-	-	-
mongola	Mnt07	hp2	OP946641	-	-	-	-	-	-
mongola	CL35m	hp1b	OP946642	-	-	-	-	-	-
mongola	CL37m	hp1b	OP946643	-	-	-	-	-	-
mongola	CL38m	hp1a	OP946644	-	-	-	-	-	-
mongola	CL54m	hp1b	OP946645	-	-	-	-	-	-
mongola	CL55m	hp4a	OP946646	-	-	-	-	-	-
mongola	CL56m	hp1b	OP946647	-	-	-	-	-	-
mongola	CL57m	hp1b	OP946648	-	-	-	-	-	-
mongola	Mnt26z	hp1a	OP946649	-	-	-	-	-	-
mongola	Mnt05	hp1b	OP946650	-	-	-	-	-	-
mongola	Mnt06	hp1b	OP946651	-	-	-	-	-	-
mongola LT	20022A03	hp1b	OP946652	-	-	-	-	-	-
sidonia	S1-22064B02	hp1b	OR178498	-	-	-	-	-	-

sidonia	S2-22064B03	hp1b	OR178499	-	-	-	-	-	-
sidonia	S3-22064B04	hp1b	OR178500	-	-	-	-	-	-
sidonia	S4-22064B05	hp1b	OR178501	-	-	-	-	-	-
croceus	CL151		OR178497	OR539270	OR539252	OR539269	-	-	-
