# Supplementary material for: Phylogeography of Two Enigmatic Sulphur Butterflies, Colias mongola Alphéraky, 1897 and Colias tamerlana Staudinger, 1897 (Lepidoptera, Pieridae), with Relations to Wolbachia Infection
Source: Insects. 2023 Dec 13;14(12):943. doi: 10.3390/insects14120943 (PMC10743618; doi:10.3390/insects14120943)
Supplement: Supplementary file 1 [file insects-14-00943-s001.zip › Table_S3.pdf]

Table S3. Variable sites of the studied *Ca-ATPase*, *CAD*, and *H3* nuclear genes fragments among the 17 sequenced samples of *C .mongola*/*C. tamerlana.*

Sample ID	COI haplotype	*CAD*																						
		13	82	145	148	151	169	178	193	211	246	247	256	298	319	342	352	406	514	541	580	685	719	763
CL42m	hp1a	C	G/T	A/G	A	A	T	T	A/C	C/T	T	A/T	A	T	A	A	A	A/G	A	C/T	G	A	G	T
Mnt17	hp1a	C	G	G	G	C	A	C	A	T	T	A	A	A	G	A	A	A	A	T	G	A	G	C/T
CL34m	hp1c	C/T	G/T	A/G	A	A	T	T	A/C	C/T	T	A/T	A/T	T	A	A	A	A/G	A/T	C/T	G	A	G	T
CL39m	hp2	C/T	G	A/G	A/G	A/C	A/T	C/T	A	C/T	T	A/T	A/T	A/T	A/G	A	A/G	A	A/T	T	A/G	A/G	G	T
CL43m	hp2	C	G	G	A/G	A/C	A/T	C/T	A/C	T	T	A	A	A/T	A/G	A	A	A	A	T	G	A	G	C/T
Mnt18	hp2	T	T	A	A	A	T	T	A	C	T	T	A	T	A	A	A	A	A	T	G	A	A	T
Ku02	hp2	C	G	G	G	C	A	C	A	T	T	A	A	A	G	A	A/G	A	A	T	A/G	A/G	G	T
Mnt28z	hp3a	C	G	G	A/G	A/C	A/T	C/T	A/C	T	T	A	A	T	A/G	A	A	G	A	C/T	G	A	G	T
CL40m	hp3a	C	G/T	A/G	A	A	T	T	A/C	C/T	T	A/T	A	T	A	A	A	G	A	C/T	G	A	G	T
Mnt04	hp3b	C	G	G	A/G	A/C	A/T	C/T	A/C	T	T	A	A	A/T	A/G	A/G	A	A/G	A	C/T	G	A	G	T
Mnt30z	hp3d	C	G	G	A	A	T	T	C	T	T	A	A	T	A	A	A	G	A	C	G	A	G	T
Ku01	hp3e	C/T	G/T	N	G	A/C	A/T	C/T	A	C/T	T	A/T	A	T	A/G	A	A	A	A	T	G	A	A	T
Mnt29z	hp3e	C	G/T	A/G	A	A	T	T	A/C	C/T	G/T	A	A	T	A	A	A	A/G	A	C/T	G	A	G	T
Mnt20	hp4a	C/T	T	A	A	A	T	T	A	C	T	T	A/T	T	A	A	A	A	A/T	T	G	A	G	T
Mnt21	hp4a	C/T	G/T	A/G	A	A	T	T	A/C	C/T	T	A/T	A/T	T	A	A/G	A	A/G	A/T	C/T	G	A	G	T
Ku03	hp4a	C	G/T	A/G	A	A	T	T	A/C	C/T	T	A/T	A	T	A	A	A	A/G	A	C/T	G	A	G	T
Ku04	hp4a	C	G	G	G	A/C	A/T	C/T	A/C	T	G/T	A	A	T	G	A	A	N	A	C/T	G	A	G	C/T

Table S3 (continuation). Variable sites of the studied *Ca-ATPase*, *CAD*, and *H3* nuclear genes fragments among the 17 sequenced samples of *C .mongola*/*C. tamerlana*.

**Sample ID**	***COI***	***Ca-ATPase***				***H3***
	**haplotype**	**238**	**352**	**379**	**406**	**46**
CL42m	hp1a	C/T	G	A	T	G
Mnt17	hp1a	C	G	A/G	C/T	G
CL34m	hp1c	C	G	A/G	C/T	C
CL39m	hp2	C	G	A	T	G/C
CL43m	hp2	C	A/G	G	C	G
Mnt18	hp2	C	G	A/G	C/T	C
Ku02	hp2	T	G	A	T	G
Mnt28z	hp3a	C	G	A/G	C/T	G
CL40m	hp3a	C	G	A/G	C/T	C
Mnt04	hp3b	C	G	A/G	C/T	G/C
Mnt30z	hp3d	C	G	A/G	T	G/C
Ku01	hp3e	C	G	A	T	G/C
Mnt29z	hp3e	C	G	A	T	G/C
Mnt20	hp4a	C/T	G	A	T	C
Mnt21	hp4a	C	G	A	T	G/C
Ku03	hp4a	C	G	A/G	C/T	G/C
Ku04	hp4a	C	G	A	T	G
